# A Research Agenda for Malaria Eradication: Modeling

**DOI:** 10.1371/journal.pmed.1000403

**Published:** 2011-01-25

**Authors:** The malERA Consultative Group on Modeling

## Abstract

Malaria modeling can inform policy and guide research for malaria elimination and eradication from local implementation to global policy. A research and development agenda for malaria modeling is proposed, to support operations and to enhance the broader eradication research agenda. Models are envisioned as an integral part of research, planning, and evaluation, and modelers should ideally be integrated into multidisciplinary teams to update the models iteratively, communicate their appropriate use, and serve the needs of other research scientists, public health specialists, and government officials. A competitive and collaborative framework will result in policy recommendations from multiple, independently derived models and model systems that share harmonized databases. As planned, modeling results will be produced in five priority areas: (1) strategic planning to determine where and when resources should be optimally allocated to achieve eradication; (2) management plans to minimize the evolution of drug and pesticide resistance; (3) impact assessments of new and needed tools to interrupt transmission; (4) technical feasibility assessments to determine appropriate combinations of tools, an associated set of target intervention coverage levels, and the expected timelines for achieving a set of goals in different socio-ecological settings and different health systems; and (5) operational feasibility assessments to weigh the economic costs, capital investments, and human resource capacities required.

## Abstract 2

The Malaria Eradication Research Agenda (malERA) Consultative Group on Modeling outline a research and development agenda to ensure that appropriate models are in place to guide malaria elimination.

**Summary Points**

- Mathematical modeling can guide all stages of malaria elimination and eradication by synthesizing information, quantifying uncertainty, and extrapolating current knowledge
- Modelers and users/stakeholders need to work closely with each other to ensure that models meet user needs and end users understand the current limitations of malaria transmission models
- A framework for modeling is being established that is both collaborative and competitive
- Models must be closely tied to all the available data, and databases and model outputs should be harmonized
- A single approach aiming at one, comprehensive model for malaria elimination/eradication has limited value; instead a variety of models and analytical approaches should be employed to guide effectively elimination efforts.

## Introduction

A global malaria eradication effort will require massive changes to a complex web of interconnected biological systems. The optimal path to eradication is intrinsically unpredictable because of the potential for parasites and vectors to evolve, the waxing and waning of human immunity, and behavioural changes in human and vector populations. The range of conditions that favour malaria transmission are so varied and diverse that decisions and plans cannot be based solely on the evidence that has been acquired in randomized control trials conducted in only a few settings. To succeed, eradication will require a strategic plan that is constantly updated with the latest surveillance, monitoring, and evaluation data. Moreover, planning processes involve some sort of conceptual model, and this model will necessarily consider many potential sources of uncertainty. Rational quantitative mathematical models provide the best way to synthesize information, quantify uncertainty, and extrapolate current knowledge. Such models can provide critical quantitative insights that are not otherwise possible.

The unique contributions that malaria modeling could potentially make to research and policy for malaria eradication led to the formation of a malERA Consultative Group on modeling tasked with defining a research and development agenda for modeling within a comprehensive malaria eradication research agenda. Our discussion about the proper use of models focused on balancing the need to provide robust policy recommendations while maintaining the energy and creativity of competitive science. The following document describes the history of malaria modeling, discusses the framework we developed for reaching consensus on the basis of independently derived models, provides an agenda to improve the science of modeling with supporting curated databases and digital interfaces, and identifies priority tasks within the broader agenda.

## Historical Background

Malaria transmission models originated with Ronald Ross during a trip to organize malaria control in Mauritius (1907–1908) [1], but the models of George Macdonald [2] were applied more systematically during the Global Malaria Eradication Program (GMEP) from 1955 to 1969 [3]. Macdonald emphasized the importance of measuring quantities that were relevant for eradication planning, such as the stability index (the expected number of human bites by a mosquito over its lifetime) and the basic reproduction ratio, *R*_0_ (the expected number of human cases that would arise from each human case in a population with no previous exposure to malaria and no malaria control) [4]. Mathematical analysis helped to explain why indoor residual spraying with DDT was such a potent malaria control strategy [5]. Later, mathematical modeling played a key role in the design and analysis of the Garki project in Nigeria [6], as well as the introduction of new indices to measure transmission, including vectorial capacity and the human blood index [7],[8].

Despite its important contributions, the overall role of mathematical modeling in the GMEP was limited. Modeling informed the design of the “attack phase” of malaria eradication [3], but not the design or implementation of other phases, and there were no provisions made to evaluate or update the design of the GMEP despite its obvious limitations. For example, the Pare-Taveta Malaria Scheme [9], which was implemented over 3.5 years in villages on the Tanzania-Kenya border, reduced malaria prevalence to less than 5%. Without sustained investments, however, malaria endemicity rebounded within 10 years of the program ending. The lessons of this and other schemes were that malaria control would require longer interventions and at a much larger scale in the African context; the implications for the broader program were never considered.

The GMEP also never considered what would happen if the initial attack phase failed. Moreover, application of the modeling was mainly limited to the Global Malaria Program in Geneva, which was not considered to be an intrinsic part of the research agenda for the GMEP. The failure to integrate and the neglect of research were partly due an emphasis on streamlining GMEP's operations and contributed to a “one-size-fits-all” mentality, with programmatic criteria based on early successes in Europe. Despite having identified DDT resistance in *Anopheles sacharovi* in 1951 [10], the only GMEP plans for dealing with resistance were to have a highly focused and time-limited program. By 1964, the GMEP had reached only approximately 3.3% of the malarious area in Africa, and the efforts were mainly concentrated at the margins of the continent [11]. After a WHO meeting in Brazzaville in 1972, formal plans for dealing with malaria in many African settings were devised [12] in which malaria elimination was not considered to be feasible, and “control” was presented as an alternative and defined as reduction of malaria to a point where it was no longer a major public health threat. A final failure of the GMEP was in not providing guidelines for establishing quantitative and operationally meaningful definitions and milestones for measuring progress towards control in a range of contexts.

The failure of the GMEP was due to many factors, including the collapse of funding [13]. However, better research with built-in monitoring, surveillance, and evaluation would have contributed to the long-term prospects for the success of the GMEP, and within that research-oriented framework, mathematical modeling could have played a pivotal role. Insufficient use of modeling was not the reason why the GMEP failed, but it could have played a stronger role in helping to anticipate, analyze, and adjust to some of the other problems that developed, such as the evolution of insecticide resistance.

Since the GMEP, substantial advances have been made in the theory and simulation modeling of malaria transmission (see Text S1), but the main research challenge for malaria eradication will be to integrate these models with surveillance, monitoring, evaluation, and with the revision of national and regional plans through every phase of eradication.

## A Consultative Framework for Malaria Modeling

After reviewing the role of malaria modeling in past control and eradication programs, the Consultative Group on modeling discussed the best way of organizing modelers and modeling. A consensus emerged that a unified approach aimed at developing an all-encompassing model for malaria elimination or eradication would probably repeat the mistakes of the past, and would therefore be inadequate. Instead, we agreed that accomplishing the modeling research agenda for eradication, avoiding errors, and providing robust advice for the future would require a framework that facilitates competitive and collaborative interactions and active communication between modelers and other scientists, research activities, surveillance, monitoring and evaluation, and that is based on a shared set of data resources.

**Figure 1:**
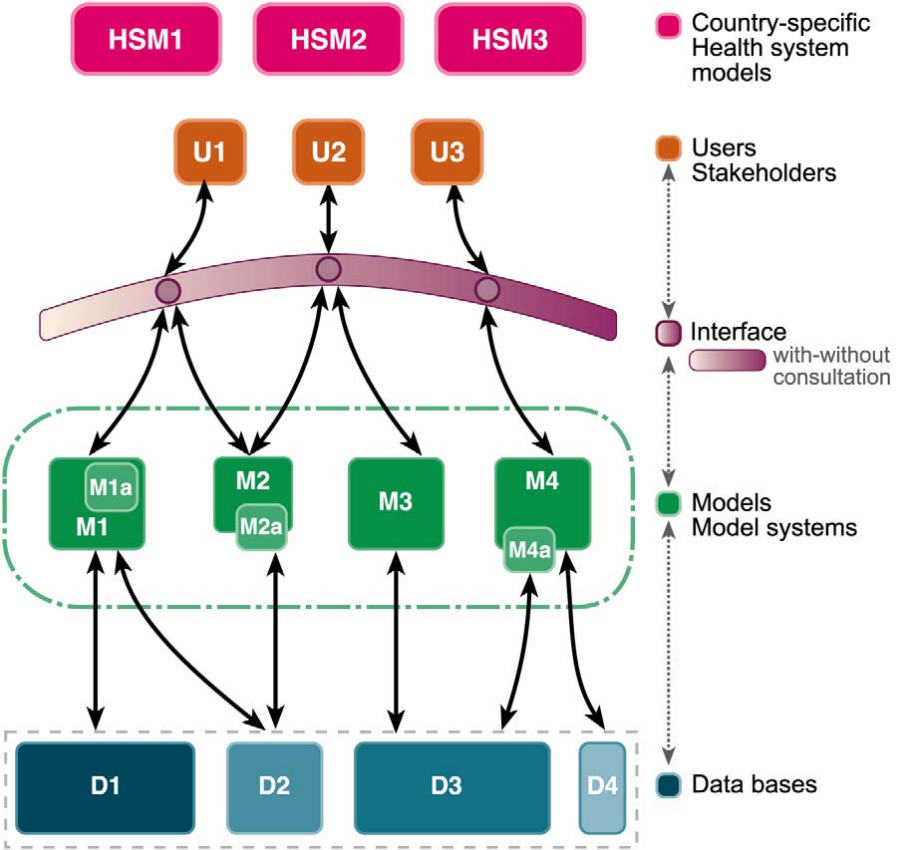
A comprehensive framework for malaria modeling. Consultations will allow policy makers, research scientists, and other stakeholders (U, users/stakeholders) from different country-specific health systems (HSM, country-specific health system models) to draw advice and analysis from multiple, independently derived models (M) grounded on data collected (D, data bases) from research on vector ecology, malaria epidemiology, and control through an interface that emphasizes direct engagement between modelers or modeling groups and end users. Image credit: Fusión Creativa.

We therefore established and endorsed a framework, motivated by climate modeling under the Intergovernmental Panel on Climate Change, which is both collaborative and competitive (Figure 1). In this framework, the core modeling functions would be conducted by independent teams, working in isolation and then coming together to compare and harmonize their results. The teams would compete with one another to provide answers to questions, and yet they would be cooperatively engaged in the common goal of finding the best solution to a defined set of problems. An added advantage of this approach, which builds on and formalizes the successful way in which scientific research ordinarily takes place, is a rapid critique from other competent modeling teams that limits the excesses of particular models or modelers and emphasizes the limitations of each approach and of models overall.

Two important features of this framework are the interface between modelers and the users, and the development of curated databases that are shared among all modelers. The Consultative Group felt that direct contact between modelers and users was the best way for modelers to be aware of the needs of their users, to be aware of new developments and data, and for modelers to communicate the limitations of their models. However, some information could be usefully shared through digital interfaces. The Consultative Group also recognized that the needs of the users would evolve over time, and that the models must be iteratively updated (the dashed arrows in Figure 1). It also regarded databases and digital interfaces as essential to the development of modeling and prioritized them as part of the research and development agenda for modeling.

Importantly, because this type of framework has not been part of the culture of malaria modeling, one of the first tasks of the modeling research and development agenda will be to operationalize the framework in Figure 1, and formally establish a process for consultations on relevant policy matters.

## The Potential Role of Modeling: Strategic Planning and Technical Feasibility Assessment

Malaria modeling should be used to inform strategic planning and malaria elimination assessments at a range of scales from global policy to local-level planning, and in guiding malaria control whether or not such activities are considered the first step towards malaria elimination. Strategic planning involves the assessment of where and when resources should be allocated to achieve elimination/eradication. Technical feasibility assessments define an appropriate combination of tools, an associated set of target intervention coverage levels, and the expected timelines for achieving reduction in burden, transmission interruption, and finally malaria elimination. Models can provide a rational and quantitative framework for integrating a range of implementation strategies, including optimizing the mix of interventions in a socio-ecological setting with different health systems to achieve a set of goals leading to malaria elimination. These results must be linked to operational assessments to describe the economic costs, capital investments, and human resource capacities required with explicit consideration of the long-term financing of malaria control and elimination.

To be of greatest benefit, models developed for malaria elimination support must specifically address changes in parasite, human, and animal hosts, and vector populations across a range of endemicities and health system conditions and capacities through the different phases of a malaria elimination program. These phases can be broadly categorized as: the initial planning phase (phase I); the introduction of interventions to interrupt transmission leading to zero incidence (phase II, which corresponds to the Global Malaria Action Plan [GMAP] “pre-elimination through elimination” phase); and “holding the line” (phase III or the GMAP “prevention of re-introduction” phase). Each phase has different goals and operational requirements, and will require different types of models. For each phase, models can be used to optimize the sequence and combinations of interventions, and for monitoring evaluation and surveillance. Although economic models and behaviour and malaria transmission models have been developed in isolation, there is a great need for models that consider transmission within economic models, and vice versa, for all phases of elimination. Models can also be used to define and test phase-specific target product profiles (TPPs) of new tools. TPPs describe the ideal, desirable, or minimally sufficient properties of a new tool in formalized documents that facilitate discussion between funding agencies, product developers, and regulatory agencies. TPPs will remain relevant throughout the path towards global eradication as endemicity and health system requirements change, and as countries adapt to their own unique challenges.

We anticipate that strategic planning will also need to account for variation in the mix of parasite species across the geographical range of malaria. At present, models are mainly focused on single-species *Plasmodium falciparum* infections and require further development for *Plasmodium vivax*, other parasite species, and mixtures of species.

### Phase I: Planning

Planning involves a technical assessment to determine whether elimination is feasible, based on the baseline distribution of malaria and current tools, and on what level of intervention coverage is required to reduce transmission intensity sufficiently to achieve elimination. A key variable here is the basic reproduction ratio *R*_0_. At a country level, it may not be possible to provide direct estimates of *R*_0_. However, several measures related to transmission intensity, including parasite prevalence, age-stratified seroprevalence, and entomological inoculation rate may be available. Mathematical models are required to translate these measures into a single comparable quantity. A likely output of such an exercise would be a map of *R*_0_ at an appropriate spatial resolution.

The technical requirements for elimination are also directly related to the operational and financial requirements for elimination, so these must be linked in models to assessments of health systems, economic costs and benefits of elimination, the risks of failure, and the likely funding.

The second aspect of an initial feasibility assessment is to define vulnerability, namely the risk that cases may be imported from surrounding malaria-endemic countries. Direct measurement of vulnerability is complicated in areas in which endemic transmission is ongoing and will only be achievable when imported cases become a substantial fraction of all cases. Preliminary assessments thus need to be estimated indirectly by taking into account patterns of endemicity in neighbouring countries and the level of cross-border movements. Spatially stratified mathematical models can aid these assessments, which are not considered in current strategic models. Each country's economic incentives to eliminate malaria may be strongly influenced by the decisions of their neighbours. The elimination of malaria from an entire region reduces the chances of re-introducing malaria and is likely to create a regional public good, which would make a strong economic case for coordinating elimination campaigns among countries.

Modeling also has a key role to play in selecting appropriate combinations of interventions to interrupt transmission and in setting response timelines and expectations of impact. Models can help to elucidate whether different interventions are likely to be synergistic, and when they can be deployed to best effect. Although insecticide-treated nets, indoor residual spraying, and artemisinin combination therapies have been used successfully in well-designed randomized control trials, these trials have been conducted in a limited number of settings and the results of applying the same control measures at the same intensity in different places may vary depending on such factors as the intensity and seasonality of transmission, the characteristics of the parasites, and the immunological status of human populations. There is no evidential basis for extending the results from existing randomized control trials to the whole range of conditions that exist in the real world and it is impossible to conduct randomized control trials that cover all the factorial combinations of those conditions. Using mathematical models, such experiments can be simulated with minimal expense on a computer to obtain immediate answers. Mathematical models are thus an indispensable tool for thinking carefully and quantitatively about the dynamics of malaria control and elimination. Although computer-based simulation studies are not a substitute for reality, they do provide a highly refined and structured way of synthesizing information and testing ideas. In particular, they provide a useful tool for testing how differences in transmission can lead to different results when the same interventions are applied in two different populations.

Finally, drug and pesticide resistance were blamed for slow progress during the GMEP and may have contributed to its failure [11]. Malaria elimination and global eradication must therefore anticipate that resistance will evolve and must incorporate this inevitability into the plans. The functional significance of drug and pesticide resistance on transmission has therefore been identified as an important research topic for modeling to facilitate effective strategic planning.

### Phase II: Pre-elimination through Elimination

The context for transmission and the operational challenges inevitably change as transmission is reduced to low levels. Previous experience unambiguously demonstrates that low-level transmission presents protracted challenges that contribute to a loss of commitment of countries and donors. In particular, the biology of *P. vivax* poses unique challenges for malaria elimination during this phase because of the dormant liver stages. Experience during the previous malaria eradication campaign suggests that *P. falciparum* will be eradicated long before *P. vivax*. The patterns of species composition are therefore critical concerns for elimination, and changes in the patterns can be used as a measure of progress towards elimination of *P. falciparum*.

As exposure to malaria declines, malaria immunity begins to wane, so each new case is more likely to result in clinical disease. During these later phases, different strategies may be deployed to shorten the response timelines, such as mass drug administration, passive or active case detection, localized outbreak control, public relations campaigns, prophylaxis for citizens traveling in malaria-endemic areas, and possibly border controls. These strategies can be supplemented by well-timed vector control. The optimal and timely use of interventions may shorten the time until elimination by decades.

Modeling can serve several roles in this phase. The first is to help set expectations about the inevitable long response timelines, since these will place increasing challenges on public health officials to justify the expense. Setting unrealistic timelines can undermine support for an elimination campaign and contribute to failure.

As malaria becomes rare, the role of monitoring, evaluation, and surveillance becomes critical [14]. Thus, a second role for modeling is to help organize information about imported malaria, to characterize transmission foci, and to design interventions. Models can be used to simulate low-level transmission and control and thus to help design and establish efficient sampling schemes appropriate for the low and declining level of endemicity.

During this phase, new programmatic skills and capabilities need to be developed that will prevent re-introduction or “hold the line” in perpetuity. Modeling can help to establish the minimal essential intervention coverage levels needed in this new transmission setting, and models can help to fine tune the programs to minimize both costs and the risk that malaria will re-establish. Another important need at this stage will be to define specific timelines and optimal strategies for *P. vivax* elimination.

As transmission becomes less intense, it also becomes more sporadic and often highly focal. In many countries, a constant flow of imported malaria can generate small clusters of ongoing transmission without the re-establishment of endemic transmission. Consequently, this is likely to be a long phase for countries or geographical areas close to malaria-endemic areas. Moreover, the accomplishments of countries or geographical areas that have eliminated their endemic reservoir and limited onward transmission but continue to have sporadic outbreaks may not be recognized. Mathematical modeling can help to describe and interpret the patterns of endemic, low-level onward transmission or imported malaria, and provide important feedback to monitoring and evaluation programs.

### Phase III: Prevention of Reintroduction

Mathematical modeling has two essential purposes once local elimination has been achieved. First, it can be used to assess the sustainability of elimination in the local area or country. Second, it provides a formal set of analytical tools to address the unique challenges of keeping malaria out of countries that have successfully eliminated the parasites.

After elimination, the basic approaches to holding the line are broadly similar to the strategies towards the end of the program for “getting to zero.” However, countries will face increasing pressure to shift resources away from malaria control to other, more pressing issues. Surveillance during this phase will remain critical, especially to identify where and when malaria is imported. In these circumstances, model development will play an important role in improving the criteria for and the process of certifying malaria elimination, and in determining when malaria elimination can be scaled back without risking re-emergence of the parasite.

The sustainability of malaria elimination is related to several factors. The evolution of drug and insecticide resistance, vaccine-escape variants, human and vector behavioural changes, and other kinds of “resistance” can threaten to undermine malaria elimination programs at every phase. Similarly, volatility in outside donor funding can threaten the viability of elimination efforts and country-level motivation. Modeling provides a realistic framework for setting donor expectations, as well as a way to anticipate the problems that might arise. Models can also be used to illustrate the consequences of stopping too early or failing to finish the job. Endgame planning is an integral part of strategic planning for regional elimination.

## Research and Development Requirements for Model Improvement

To best support the specific goals of malaria elimination, a research and development agenda is required to improve modeling. Several topics are currently in need of additional model development and the acquisition of key pieces of evidence. Some of these topics have only recently been identified by research, others have not been addressed because they are considered to be of limited interest.

### Biology and Natural History

As the complex life cycle of malaria parasites becomes better understood, new and improved models are needed to make use of this information in elimination programs. First, better models of the development of parasite species in their human and vector hosts need to be devised and the features of the parasite life cycle need to be quantified better. In particular, there is a need for better data and models to quantify the importance of relapse in *P. vivax* and the importance of other unique aspects of non-falciparum parasites, and to quantify the nature of interactions among all species [15].

Models are also needed to capture the human infectious reservoir across a range of transmission intensities. Ill-understood factors contribute to variability in the transition rates of parasites from the asexual stage onwards and through each subsequent stage of the transmission cycle in people and mosquitoes. Even if for operational purposes, individuals with measurable parasites are considered to be infected and therefore not distinguished from gametocyte carriers, it remains important to capture the relative infectiousness of different population groups in models.

The abiotic determinants of mosquito densities and the dynamics of larval stages are poorly understood. Thus, there is a need for models that consider the effects of, for instance, seasonality and dry season refuges. Such models can provide information about the potential of larval control and optimal larval control strategies. The effects of infection and environment on adult mosquito behaviour, infectivity, and survival also need to be considered in modeling efforts [16].

The existence of natural immunity to malaria that partially protects against disease or reduces transmission is a particularly challenging problem for epidemiological models. The stimulation, duration, and effects of acquired immunity need to be better understood, and this understanding must be incorporated into models to determine, for example, how many years of zero transmission must pass before symptomatic disease can be used as a marker of re-introduction [14],[15].

Another aspect of parasite natural history that is not comprehensively addressed in current malaria models is heterogeneity in hosts, parasites, and vectors. Substantive problems in measuring levels of heterogeneity need to be addressed and these effects need to be appropriately incorporated in models. Heterogeneity is likely to have a greater impact on model results as transmission is reduced.

Finally, as transmission is reduced, the effects of geographical movement of the parasite that occur because of both vector and human movements will dominate the dynamics. The relative role of movement versus dry-season refuge in maintaining the infectious reservoir in epidemic settings remains poorly understood but will be a major determinant of the required control strategy to achieve elimination and hold the line. Human movement in particular is difficult to quantify on the basis of current data. Spatially explicit models will need to be developed that can adequately capture parasite movement and the linking of spatially distinct populations [15].

### Effects of Interventions

Models of the dynamics of drugs (pharmacokinetics and pharmacodynamics [PK/PD], dosing regimens) and of vaccines that interrupt transmission at various stages need to be developed. In addition, there is a need to develop models that describe the ecology of genetically modified mosquitoes and the potential impact of such insects on malaria transmission [2],[16].

The scope of models needs to be expanded to consider the overall effects on and of health systems and to account for the capabilities of preexisting health system infrastructures. Modeling needs to include the effects of combinations of interventions/tools and the effects of scheduling of interventions. It also needs to support the optimization of TPPs and their alignment with the existing packages of interventions. All these components need to be supported by microeconomic appraisal [17].

### Effects of Interventions on the Evolution of Resistance

Resistance to interventions is broadly defined to include any heritable changes that reduce the effectiveness of drugs, pesticides, vaccines, and other interventions. TPPs need to be considered prospectively with model-based analyses of the likely evolution of resistance. Modeling approaches need to be developed that integrate population genetics and direct intervention effects, such as PK/PD data for drug resistance, behavioural and physiological changes in response to vector control, and molecular epidemiology for vaccine escape variants. A critical feature for models is better characterization of the biological cost of resistance. As new tools are developed, it will be important to plan deployment strategies with an awareness of the effects they will have on the evolution of resistance [16],[18],[19].

## Prerequisites for Achieving Modeling Objectives

To achieve these modeling objectives and to answer specific research and operational questions, there is a need to create, curate, and harmonize databases. An interface and a supporting infrastructure (see Figure 1) must also be created to facilitate combining databases and diverse datasets, including those that will be generated by mathematical modeling. Importantly, as much information as possible should be openly accessible from a single place to facilitate modeling and the dissemination of model outputs to the broader community of users, stakeholders, and contributors. Below we discuss the perquisites for achieving modeling objectives.

### Compilation and Curation of Databases and Harmonization of Model Outputs

The purpose of the databases will be to collect information for various users in one place. For modelers, this information is required to parameterize and validate malaria models, and to extend them geographically and temporally. The malaria community requires more general information for monitoring and evaluating progress towards control/elimination/eradication.

A Web site that links to relevant information already on the Web, that hosts databases and appropriate interfaces to databases that are not hosted elsewhere, and that provides technologies that allow other software applications to access the hosted information will facilitate Web-based information exchange. Such a Web site would also include automatically generated information summaries and post synthetic data, data summaries, and summary statistics.

Data to be included on such a Web site should comprise, among other things, disaggregated data on the natural history of different human malaria species, disaggregated malariological field data from published and unpublished field research studies, data aggregations from searches of published and unpublished literature, and data from model outputs. The results of basic laboratory research, data on nonhuman malarias, and genomic data should be excluded from the early stages of the structure, however, except through hyperlinks to major data repositories. There should be links to relevant nonmalaria databases (e.g., UN demographic data, Demographic and Health Surveys, climate, population, and remotely sensed environmental data), but the platform should not host these databases unless this is essential for the analysis of hosted core data. Table 1 in Text S2 represents an initial list of the databases that might be hosted or otherwise harmonized. The challenges and requirements for achieving this are outlined below.

### Primary Databases and Key Models

The potential database resources in Text S2 are necessarily incomplete, but should be gradually extended and more effectively interlinked. Different modeling approaches have common data needs, many of which will be satisfied by the datasets listed in Text S2. Spatially specific data will be required by some types of models so many of these data need to be geolocated. An important subset of data is the results of malariological field studies, especially field trials of interventions; the results of observational studies (e.g., of drug action) are also important. Parasitological data that are specifically required include infectious durations and data from field studies that can be used to estimate clearance rates. Specific entomological data requirements include data on vector survival, behaviour, and biting rates (including heterogeneity in biting rates). There will be a need to include global databases of weather and climate data, in particular temperature, rainfall, humidity, and soil moisture. New databases will need to be developed to support tracking of larval habitats and prediction of vector emergence rates. Modeling will also need to be supported by access to human demographic databases, including those of population distribution, age structure, and migration rates. This information will require access to data on transport networks (e.g., roads) and communications networks such as cell phones.

The compendium of resources detailed in Text S2 contains information sources, at various levels of complexity and in various states of assembly, that are of variable use to the modeling teams. Text S1 describes the history of modeling and the range of models currently available and under development.

### Minimal Reporting Standards

Databases without descriptors are a static resource. A traditional, if not widely used, way to audit data resources is to describe them in a peer-reviewed article and append the information as supplementary material. A new publication route for data, such as an entirely new journal or a new article style in existing journals, is perhaps required, with the intention of encouraging the release of preexisting unpublished data while solving the problem of suitable accreditation for data sources.

### Data and Model Curation and Sustainability

The curation and improvement of large databases requires significant personnel capacity for correction and assembly of new information. Furthermore, this capacity needs to be sustained in the long term for its value to remain and agreement has to be reached on what constitutes acceptable information quality, how to define it, and how to moderate correction. All stakeholders, not just researchers, must be made aware of the limitations of models and the data on which they rely. Data and model curation needs to be inclusive while flagging and addressing known problems and using disclaimers to avoid excessive reliance on questionable information.

### Common Ontologies, Frameworks, and Metadata Standards

An evolving way to audit database resources is to provide machine-readable metadata so that third parties can employ Web services to seamlessly harvest and/or integrate database information in downstream applications. This harmonization process requires that all databases be accessible to the extent that they can be shared at the human and machine level with any third party with as little administrative, technical, and logistical support as possible. This prerequisite is rooted in the concept of the semantic web, which provides the methods and technologies that allow machines to understand, share, and reuse data in real time across application, enterprise, and community boundaries. There will be many benefits in investing in a semantic web, not least the availability of resources that can be updated, minimizing human errors in translation for third-party applications.

To formalize minimum standards in databases, an ontology is often specified. An ontology is defined as relationships among a set of terms in an agreed nomenclature that describe a database resource. There are many examples of ontologies, all tailored to specific applications. To develop an ontology for our specific purposes (if it were considered valuable), the most relevant existing ones could be reviewed, a hybrid ontology of useful descriptors constructed, and an expert group established to fill the gaps. Ontologies are critical for translating minimum reporting requirements into machine-readable metadata. However, paradoxically, several metadata “standards” are under development. Advice should be solicited from the information technology community on which to adopt. Finally, candidate models may require some minor modifications to their outputs for harmonization with other similar models. This task could be done by the original authors of the model or they could provide the necessary information and a mandate for the modification to be performed by the curators.

### Incentives for Data Sharing

Proper incentives are required to guarantee that the data-sharing tasks will be accomplished. Data provision and model integration are challenging tasks that do not achieve immediate recognition but facilitate exciting science and improve public health impact at some future point. To implement semantic enrichments to databases and make models more widely accessible will take time, thought, and energy. Individuals and groups should be incentivized to do this on new and also, importantly, valuable old datasets. This process will require mechanisms for attribution, quality, and provenance control, and long-term curation and hosting obligations. There is a need to decide how resources will be partitioned between existing databases and downstream resources/portals.

Open-access data sharing is a collective benefit that outweighs individual concerns, even though most of the communities gathering relevant data do not yet operate a culture of open access. Accordingly, access to data needs to be negotiated carefully, with the general philosophy being to minimize restrictions and to gradually negotiate wider access for sensitive datasets as questions of ownership and attribution are resolved.

### Accessing Software-Engineering Skills

At present the level of interaction between end users of model results and those developing and implementing the models is relatively limited. The creation of interfaces that allow user access depends on computer scientists and programmers and they must work closely with domain experts to ensure that interfaces meet the needs of all stakeholders. Most institutes carrying out malaria research have only limited capacity to develop Web database applications. Professional software teams with close links to malariologists are needed to set up and maintain such a system.

### Interface for Users and Stakeholders

There are a wide range of potential end users of mathematical models and their outputs, including other researchers, funding bodies, program implementers, planners, and policymakers. All of these end users have different needs in regards to the models, and there are many ways in which they could potentially interact with them. The most common and effective interface is the modeler, who will ideally be embedded in a research or policy-making network with the research scientists, medical doctors, public health officials, or policy makers who will be using the models. Such an interface would facilitate active and direct communication about models and outputs, alert modelers to the availability of new data, and keep modelers current with a changing situation, which would lead to iterative updating of models. Direct contact with modelers can be supplemented in specific cases so that a researcher or policymaker is able to interact directly with a computer to obtain information ranging from specific queries about a predefined set of scenarios to more sophisticated outputs using decision-support systems. Regardless of the level of contact, it is important that stakeholders are engaged throughout the model development process so that model outputs and interfaces match user needs, and end users understand the current limitations of transmission models, in particular in terms of making quantitative predictions.

There is currently no readily available interface or “cyberinfrastructure” that brings together data, models, and stakeholders seamlessly at the required scale and scope, although prototype systems are being tested. The description below outlines what is feasible in the short term, assuming sufficient research and development support. Text S3 provides a more detailed design.

Given a (possibly distributed) annotated database, a set of software models with well-defined application programmer interfaces (APIs), and a semantic web or ontology, it may be possible to develop a cyberinfrastructure that automates much of the deductive reasoning required to answer common stakeholder-specified questions. Models can be fitted into well-established paradigms for data search and integration. The cyberinfrastructure would translate these questions into appropriate analyses on the model output. Missing data inputs could be replaced by data from other similar settings as extracted from the underlying databases with the appropriate caveats made clear to the end user. A question may trigger a cascade of data retrieval and model execution, all managed by the cyberinfrastructure. If the available data and models are insufficient to answer the question, the gaps would be noted to assist in research program development. Output of analyses performed by the structure would include a comprehensive list of citations of the source materials. A list of caveats to data inputs or model outputs (provided by the stakeholders) on the scope of appropriate use would also be included. The cyberinfrastructure therefore provides those contributing data and developing models with an incentive to include their information in the system with the assurance that results will not be misinterpreted.

## Conclusions

On the basis of our discussions, we propose a research and development agenda for modeling that will effectively support operations and important research questions in attempts to achieve elimination and eradication of malaria and that lists the prerequisites and research questions for the development of modeling based on a comprehensive framework (Box 1). A single approach aiming at one, comprehensive model for malaria elimination/eradication has limited value. Rather, we will profit at the operational level as well as at the scientific level from answering the research questions and issues as outlined in this paper using a variety of models and analytical techniques, supported by direct interactions with modelers and common user interfaces, and linked to curated essential databases.

**Box 1. Research and Development Agenda for Modeling**

**Modeling approaches to guide elimination and eradication**

- To provide practical tools to help planners and policymakers assess the technical, operational, and financial feasibility of malaria elimination.
- To assist in optimizing combined interventions for elimination in different transmission and health systems contexts.
- To assess and optimize TPPs for interventions and for monitoring and evaluation, and to determine the potential contribution of the products to the different phases of malaria elimination.
- To ensure flexible management in choosing and designing interventions, and for designing surveillance in collaboration with monitoring and evaluation programs to identify cost-effective strategies to shorten elimination timelines.

**Further development of models and model systems**

- Further basic modeling research is required on the within-host dynamics of *Plasmodium* infections, the human infectious reservoir, bionomics and ecology of the vectors, dynamics of the stimulation and decay of human immunity, heterogeneities in hosts, vector, and parasite dynamics, and host and vector movements, to enable the models to better answer strategic questions for malaria elimination.
- Further development is required of models of drug dynamics, vaccines that interrupt malaria transmission, and the ecology of genetically modified mosquitoes. Health system attributes need to be integrated into current models for packages of interventions and linked to microeconomic outputs.
- Models need to be further developed to consider the likely development and impact of drug and pesticide resistance at the various stages of elimination across different transmission settings.

**Enabling technologies**

- Harmonization of databases and model outputs, which entails:
  - Identifying key data needs and deciding whether existing information is of sufficient quality to inform the modeling.
  - Identifying technologies that support machine-level exchange of malariometric data.
  - Recognizing the importance of creating and maintaining thoroughly annotated databases and models, along with software tools and well-documented user interfaces with close collaboration between software engineers and malariologists to support model and data curation and access.
- Development of cyberinfrastructures to generate and execute efficient workflows for answering strategic questions. Cyberinfrastructures would identify and retrieve data from distributed databases; identify and execute appropriate models, compose data, and model results across multiple spatiotemporal scales and domains; and manage information about provenance, citations, and assumptions.

## Supporting Information

Text S1 History of malaria modeling and models currently available.
(0.21 MB DOC)

Text S2 Harmonization of databases; annex of existing databases.
(0.27 MB DOC)

Text S3 Interface for users and cyberinfrastructure.
(0.18 MB DOC)

## Abbreviations

GMEP: Global Malaria Eradication Program
TPP: target product profile
