# Supplementary material for: A Research Agenda for Malaria Eradication: Modeling
Source: PLoS Med. 2011 Jan 25;8(1):e1000403. doi: 10.1371/journal.pmed.1000403 (PMC3026697; doi:10.1371/journal.pmed.1000403)
Supplement: Text S1 — History of malaria modeling and models currently available. (0.21 MB DOC) [file pmed.1000403.s001.doc]

**Annex 1. History of malaria modeling**

Throughout history, mathematical models have been used for both basic and applied purposes. After Ronald Ross demonstrated that mosquitoes transmit malaria, he developed a mathematical analysis to relate mosquito flight distances and densities under larval mosquito control [1]. After a trip to organize malaria-control activities in Mauritius, Ross published the first malaria transmission model [2,3]. Three years later, he published a new differential equation model [4,5,6,7,8,9] that is more widely used. Ross was interested in mosquito density as a cause of variation in the parasite rate [10]. His models and analysis provided a quantitative framework and justification for larval source management that dominated the first 50 years of malaria control.

In the aftermath of World War II, the discovery of DDT and the mass production of chloroquine led to a burgeoning optimism about malaria. A watershed moment came in 1950 at the Conference on Malaria in Equatorial Africa held in Kampala, Uganda, where a recommendation was made “to governments responsible for the administration of African territories that malaria should be controlled by modern methods as soon as feasible, whatever the original degree of endemicity, and without awaiting the outcome of further experiments[11].” The World Health Congress voted in 1955 to eradicate malaria, and WHO coordinated a Global Malaria Eradication Program (GMEP) based largely on indoor residual spraying with DDT [12].

About this time, George Macdonald took the first steps towards testing Ross’s theory with epidemiological [13] and entomological [14] field data. Field trials with DDT in the early 1950s had demonstrated that it was an effective way to interrupt malaria transmission. Macdonald’s analysis helped to explain that DDT and other contact pesticides worked because they severely reduced the number of mosquitoes that would live long enough to survive sporogony and transmit malaria [15].

One of Macdonald’s biggest contributions to malaria theory was his emphasis on defining and measuring quantities that were operationally relevant for eradication: the stability index and the basic reproductive numbers for malaria. Macdonald described a formula for R_0_ and clearly explained the entomological basis for measuring it [16,17]. During the GMEP, mathematical models were used for other purposes, such as establishing realistic response timelines [18]. Macdonald’s influence on the technical aspects of eradication is evidenced by his being elected to chair the committee that wrote the first technical report on malaria eradication [19].

By 1970, progress towards eradication had slowed for many reasons, including competing public health priorities and donor fatigue, DDT resistance and controversy about the environmental effects of DDT [20]. The agenda for eradication began to shift towards stable endemic control. Meanwhile, a large-scale malaria control project was launched in Garki, Nigeria, to evaluate whether malaria could be controlled in an African context with multiple, integrated interventions. In planning the Garki project, a new mathematical model was developed [21] that was innovative in several ways. Firstly, it considered superinfection, an idea that had been explored mathematically before [22,23]. Second, it considered the development of immunity in two stages: after being infected with malaria, semi-immune individuals would be less likely to test positive for malaria, and they would clear infections faster. Third, it considered a kind of transmission-blocking immunity: individuals were infectious only if they were non-immune and recently infected. One advantage of the Garki model was that it reproduced the age-specific patterns in malaria prevalence, at least qualitatively.

International donor funding for malaria control sharply declined after 1963 and remained low throughout the 1970s [24], but theoretical studies continued to explore the ideas that had been described in the Garki project, most notably superinfection. Walton had been the first to describe a formula for distribution of the MOI (multiplicity of infection) [22]. A few years later, Macdonald developed a model for simulating complex infections [23], but while the written description of his assumptions was consistent with Walton’s model, the equations actually corresponded to a different set of assumptions [25]. Bailey described a queuing model for the full dynamics of superinfection with an infinite number of genotypes using the same assumptions as Walton [26], and this queuing model had motivated the development of the Garki model [21]. The queuing model was later extended and generalized by Nåsell to describe superinfection with a finite number of types [27], and by Dietz to consider density dependence [28].

During the 1980s, motivated in part by the outcome of the Garki project, but also by studies of sexually transmitted diseases, new attention focused on the biting behaviour of mosquitoes and its consequences for disease transmission, and in particular on the importance of heterogeneous biting [29,30,31]. Mathematical modeling suggested that heterogeneous biting would amplify transmission, raising R_0_ proportional to the squared coefficient of variation of the human biting rates.

In the 1980s, May and Aron presented a new model for malaria that was motivated by the Garki model. In the Garki model, most infected individuals were not infectious (i.e. the only infectious class was *y_1_*), whether or not they had become semi-immune. Several infected classes from the Garki model could be combined together, since they were not infectious, to model malaria as an SIRS (susceptible-infected-recovered-susceptible) compartment model, with dynamics much like flu [32,33]. Later, this model was extended to consider the transmission dynamics and epidemiology of serial infection and immunity to infection with multiple parasite “strains” [34,35], and the evolution of antimalarial drug resistance [36]. The assumption in the Garki model that made these SIRS models relevant was that only non-immune individuals who had been recently infected were infectious. A critique of the Garki model is that the assumptions about immunity were very severe and not consistent with data. For example, in an area where malaria prevalence was above 90% in children, and where immunity in adults would tend to be quite high, adults did transmit malaria to mosquitoes, albeit less efficiently [37], and the patterns are broadly inconsistent with studies that identify the infectious reservoir of malaria [38,39,40,41,42,43].

To address these shortcomings, the Garki model has been reformulated to generalize the notion of immune stages and to describe stage-structured immunity in a far more general way. Stage structure deals with categorical descriptions of malaria age, and it models the transitions between them. In one simple example, the Garki model was modified to suggest that semi-immune individuals remain infectious to malaria but transmit malaria with lower efficiency[44,45,46].

The availability of high-speed desktop computing ushered in a new class of complex individual-based simulation models [47,48]. These models were based on within-host models of malaria that were calibrated to the records of infection in malaria therapy patients that described the first wave of infections, the time course of a simple infection [49,50,51,52,53], the conversion from merozoites to gametocytes [54,55] and gametocyte dynamics [56]. Such models have been extended to create simulations of both transmission and health outcomes parameterized using many distinct field measures of malaria epidemiology [48].

The GMEP was launched because malaria was a major public health burden, but from 1950 onwards research in malaria was driven by the goals of eradication with a sharp focus on interrupting malaria transmission. Malaria immunity was clearly important in areas of high malaria transmission intensity, but a quantitative picture of malaria burden and its relation to malaria transmission intensity was largely ignored until quite recently. In the 1990s, a series of studies took a more rigorous approach to defining severe malaria, and a more quantitative approach to estimating the burden of the disease. Those studies identified three generalized, overlapping disease syndromes associated with severe malaria [57,58]. Subsequent studies described different age-specific patterns for cerebral malaria and severe anemia [59,60,61,62,63]. The analysis suggested that the highest burdens might occur at intermediate transmission intensity, raising the question of whether reducing it could perversely raise the burden [64]. Other studies concluded that the burden of malaria always increases with transmission intensity [65,66]. Malaria is particularly severe in prima gravidae pregnancies, with several adverse consequences including low birth weights with different epidemiology in areas of low and high transmission [67,68,69,70,71]. These studies have raised a new set of questions about the relationship between transmission intensity and the burden of malaria that have not been explored in great detail but are starting to be addressed by models [38,72,73].

Concerns about global warming have focused some attention on the role of temperature and rainfall on malaria transmission and the potential for malaria to expand its range [74,75]. Temperature affects the rate of parasite development in the mosquito [14] and hence the boundary of climatic suitability for malaria [76,77]. In places where malaria is not controlled, though, the density of mosquitoes is a dominant source of variability in malaria transmission, in part, because of the great potential for exponential growth of mosquito populations in their larval habitat [78,79]. Over some temperature ranges, temperature plays a role in this by modifying the rate of larval development in their aquatic habitats; however, seasonal fluctuations in malaria transmission are more often driven by the relationship between rainfall and mosquito density (for example, see [80,81,82]). The effects of temperature must, however, be considered in light of other large factors at work in malaria [83].

Ironically, the first quantitative study of malaria control focused on the spatial dynamics of transmission and control [1], but the spatial dynamics of malaria remained largely unexplored by theorists for 75 years. In his 1982 book about malaria, Bailey wrote down equations that described the spatial dynamics of malaria as a set of reaction-diffusion equations [26]. Later, that work was extended to consider explicitly spatial simulations [84] and malaria dynamics in a set of heterogeneous patches linked by mosquito migration – the flux of mosquitoes across an arbitrarily defined patch border was a crude approximation to reaction-diffusion equations [85]. Most recently, the metapopulation dynamics of malaria were described and included human and mosquito migration [86].

The past decade has seen a tremendous upsurge in modeling malaria interventions, which represents a substantial advance beyond Macdonald’s landmark analysis of DDT. In his original paper, Macdonald argued that DDT would be highly effective because it substantially reduced the number of older mosquitoes that had lived long enough to transmit malaria. Macdonald did not, however, attempt to estimate a quantitative effect size associated with DDT, and his analysis probably underestimated the real effect sizes [87]. Insecticide-treated nets (ITNs) have effects similar to DDT by protecting people who use the intervention and reducing transmission overall [88]. In the past few years, a new class of models has been developed to address these concerns and to estimate the transmission effect sizes associated with ITNs as a function of effective coverage, i.e. the fraction of a population that owns and uses a net [89,90]. Similar analysis has been done to estimate the transmission effect sizes of antimalarial drugs in high- and low-intensity settings, depending on the fraction of the malaria incidence that is followed by appropriate treatment [91,92].

Without doubt, modeling – notably the recognition in the Ross/MacDonald formula of the powerful impact of vector-based interventions – strongly influenced the design of previous successful intervention campaigns. With improved quantitative and mechanistic understanding of parasite, vector and host biology, and of environmental and climatic variables, combined with the massive leap in computing power available now and in the future, it is abundantly clear that modeling has a key role to play in the design of rational intervention strategies at the individual, population, and global levels. Key steps in modeling to date are illustrated below.

**References**

1. Ross R (1905) The logical basis of the sanitary policy of mosquito reduction. Science 22: 689-699.

2. Ross R (1908) Report on the prevention of Malaria in Mauritius. London: Waterlow and Sons Limited.

3. Waite H (1910) Mosquitoes and Malaria. A Study of the Relation between the Number of Mosquitoes in a Locality and the Malaria Rate. Biometrika 7: 421--436.

4. Ross R (1911) The prevention of malaria. London: John Murray.

5. Lotka AJ (1923) Contributions to the analysis of malaria epidemiology. II. General part (continued). Comparison of two formulae given by Sir Ronald Ross. Amer J Hyg 3 (Suppl. 1): 38-54.

6. Lotka AJ (1923) Contributions to the analysis of malaria epidemiology. III. Numerical part. Amer J Hyg 3 (Suppl. 1): 55-95.

7. Lotka AJ (1923) Contributions to the analysis of malaria epidemiology. I. General part. Amer J Hyg 3 (Suppl. 1): 1-36.

8. Lotka AJ (1923) Contributions to the analysis of malaria epidemiology. V. Summary Amer J Hyg 3 (Suppl. 1): 113--121.

9. Lotka AJ, Sharpe FR (1923) Contributions to the analysis of malaria epidemiology. IV. Incubation lag. Amer J Hyg 3 (Suppl. 1): 96--112.

10. MalERA (2010) A Research Agenda for Malaria Eradication: Basic Science and enabling Technologies. PLoS Medicine in press.

11. Dobson MJ, Malowany M, Snow RW (2000) Malaria control in East Africa: the Kampala Conference and the Pare-Taveta Scheme: a meeting of common and high ground. Parassitologia 42: 149-166.

12. Najera J, Gonzalez-Silva M, Alonso PL (2010) Lessons from the past Global Malaria Eradication Program (1955-1969). PLoS Medicine in press.

13. Macdonald G (1950) The analysis of malaria parasite rates in infants. Trop Dis Bull 47: 915-938.

14. Macdonald G (1952) The analysis of the sporozoite rate. Trop Dis Bull 49: 569-586.

15. Macdonald G (1956) Epidemiological basis of malaria control. Bull World Health Organ 15: 613-626.

16. Macdonald G (1955) The measurement of malaria transmission. Proc R Soc Med 48: 295-302.

17. Macdonald G (1956) Theory of the eradication of malaria. Bull World Health Organ 15: 369-387.

18. Macdonald G, Göeckel GW (1964) The malaria parasite rate and interruption of transmission. Bull World Health Organ 31: 365-377.

19. W.H.O. (1957) Expert Committee on Malaria, Sixth Report. Geneva, Switzerland: World Health Organization.

20. Gramiccia G, Beales PF (1988) The recent history of malaria control and eradication. In: Wernsdorfer W, McGregor I, editors. Principles and Practice of Malaria. Edinburgh: Churchill Livingstone. pp. 1335-1378.

21. Dietz K, Molineaux L, Thomas A (1974) A malaria model tested in the African savannah. Bull World Health Organ 50: 347-357.

22. Walton GA (1947) On the control of malaria in Freetown, Sierra Leone. I. *Plasmodium falciparum* and *Anopheles gambiae* in relation to malaria occurring in infants. Annals of Tropical Medicine and Parasitology 41: 380-407.

23. Macdonald G (1950) The analysis of infection rates in diseases in which superinfection occurs. Trop Dis Bull 47: 907-915.

24. Najera J (1999) Malaria Control: Achievements, Problems, and Strategies. WHO.

25. Fine PEM (1975) Superinfection -- a problem in formulating a problem Tropical Diseases Builletin 75: 475-488.

26. Bailey NTJ (1982) The biomathematics of malaria. Oxford: Oxford University Press.

27. Nåsell I (1986) On superinfection in malaria. IMA J Math Appl Med Biol 3: 211-227.

28. Dietz K (1988) Density-dependence in parasite transmission dynamics. Parasitol Today 4: 91-97.

29. Dietz K (1980) Models for vector-borne parasitic diseases. Lecture Notes in Biomathematics 39: 264--277.

30. Dye C, Hasibeder G (1986) Population dynamics of mosquito-borne disease: effects of flies which bite some people more frequently than others. Trans R Soc Trop Med Hyg 80: 69-77.

31. Hasibeder G, Dye C (1988) Population dynamics of mosquito-borne disease: persistence in a completely heterogeneous environment. Theor Popul Biol 33: 31-53.

32. Aron JL, May RM (1982) The population dynamics of malaria. In: Anderson RM, editor. Population Dynamics and Infectious Disease. London, UK: Chapman and Hall. pp. 139--179.

33. Aron JL (1988) Mathematical modeling of immunity to malaria. Mathematical Biosciences 90: 385--396.

34. Gupta S, Day KP (1994) A theoretical framework for the immunoepidemiology of Plasmodium falciparum malaria. Parasite Immunol 16: 361-370.

35. Gupta S, Trenholme K, Anderson RM, Day KP (1994) Antigenic diversity and the transmission dynamics of *Plasmodium falciparum*. Science 263: 961-963.

36. Koella JC, Antia R (2003) Epidemiological models for the spread of anti-malarial resistance. Malar J 2: 3.

37. Githeko AK, Brandling-Bennett AD, Beier M, Atieli F, Owaga M, et al. (1992) The reservoir of *Plasmodium falciparum* malaria in a holoendemic area of western Kenya. Trans R Soc Trop Med Hyg 86: 355-358.

38. Drakeley CJ, Akim NI, Sauerwein RW, Greenwood BM, Targett GA (2000) Estimates of the infectious reservoir of Plasmodium falciparum malaria in The Gambia and in Tanzania. Trans R Soc Trop Med Hyg 94: 472-476.

39. Haji H, Smith T, Charlwood JD, Meuwissen JH (1996) Absence of relationships between selected human factors and natural infectivity of Plasmodium falciparum to mosquitoes in an area of high transmission. Parasitology 113 ( Pt 5): 425-431.

40. Haji H, Smith T, Meuwissen JT, Sauerwein R, Charlwood JD (1996) Estimation of the infectious reservoir of Plasmodium falciparum in natural vector populations based on oocyst size. Trans R Soc Trop Med Hyg 90: 494-497.

41. Killeen GF, Ross A, Smith T (2006) Infectiousness of malaria-endemic human populations to vectors. Am J Trop Med Hyg 75: 38-45.

42. Pethleart A, Prajakwong S, Suwonkerd W, Corthong B, Webber R, et al. (2004) Infectious reservoir of Plasmodium infection in Mae Hong Son Province, north-west Thailand. Malar J 3: 34.

43. Schneider P, Bousema JT, Gouagna LC, Otieno S, van de Vegte-Bolmer M, et al. (2007) Submicroscopic Plasmodium falciparum gametocyte densities frequently result in mosquito infection. Am J Trop Med Hyg 76: 470--474.

44. Klein EY, Smith DL, Boni MF, Laxminarayan R (2008) Clinically immune hosts as a refuge for drug-sensitive malaria parasites. Malar J 7: 67.

45. Filipe JA, Riley EM, Drakeley CJ, Sutherland CJ, Ghani AC (2007) Determination of the processes driving the acquisition of immunity to malaria using a mathematical transmission model. PLoS Comput Biol 3: e255.

46. Chiyaka C, Garira W, Dube S (2007) Transmission model of endemic human malaria in a partially immune population. Mathematical and Computer Modelling 46: 806--822.

47. McKenzie FE, Bossert WH (2005) An integrated model of *Plasmodium falciparum* dynamics. J Theor Biol 232: 411-426.

48. Smith T, Killeen GF, Maire N, Ross A, Molineaux L, et al. (2006) Mathematical modeling of the impact of malaria vaccines on the clinical epidemiology and natural history of *Plasmodium falciparum* malaria: Overview. Am J Trop Med Hyg 75: 1-10.

49. Molineaux L, Diebner HH, Eichner M, Collins WE, Jeffery GM, et al. (2001) Plasmodium falciparum parasitaemia described by a new mathematical model. Parasitology 122: 379-391.

50. Anderson RM, May RM, Gupta S (1989) Non-linear phenomena in host-parasite interactions. Parasitology 99 Suppl: S59-79.

51. Saul A (1998) Models for the in-host dynamics of malaria revisited: errors in some basic models lead to large over-estimates of growth rates. Parasitology 117 ( Pt 5): 405-407; discussion 409-410.

52. McKenzie FE, Bossert WH (1997) The dynamics of Plasmodium falciparum blood-stage infection. J Theor Biol 188: 127-140.

53. Molineaux L, Dietz K (1999) Review of intra-host models of malaria. Parassitologia 41: 221-231.

54. Diebner HH, Eichner M, Molineaux L, Collins WE, Jeffery GM, et al. (2000) Modelling the transition of asexual blood stages of Plasmodium falciparum to gametocytes. J Theor Biol 202: 113-127.

55. McKenzie FE, Bossert WH (1998) The optimal production of gametocytes by Plasmodium falciparum. J Theor Biol 193: 419-428.

56. Eichner M, Diebner HH, Molineaux L, Collins WE, Jeffery GM, et al. (2001) Genesis, sequestration and survival of Plasmodium falciparum gametocytes: parameter estimates from fitting a model to malariatherapy data. Trans R Soc Trop Med Hyg 95: 497-501.

57. Marsh K, English M, Crawley J, Peshu N (1996) The pathogenesis of severe malaria in African children. Ann Trop Med Parasitol 90: 395-402.

58. Marsh K, English M, Peshu N, Crawley J, Snow R (1996) Clinical algorithm for malaria in Africa. Lancet 347: 1327-1328; author reply 1328-1329.

59. Newton CR, Warn PA, Winstanley PA, Peshu N, Snow RW, et al. (1997) Severe anaemia in children living in a malaria endemic area of Kenya. Trop Med Int Health 2: 165-178.

60. Gupta S, Snow RW, Donnelly C, Newbold C (1999) Acquired immunity and postnatal clinical protection in childhood cerebral malaria. Proc Biol Sci 266: 33-38.

61. Gupta S, Snow RW, Donnelly CA, Marsh K, Newbold C (1999) Immunity to non-cerebral severe malaria is acquired after one or two infections. Nat Med 5: 340-343.

62. Reyburn H, Mbatia R, Drakeley C, Bruce J, Carneiro I, et al. (2005) Association of transmission intensity and age with clinical manifestations and case fatality of severe Plasmodium falciparum malaria. JAMA 293: 1461-1470.

63. Okiro EA, Al-Taiar A, Reyburn H, Idro R, Berkley JA, et al. (2009) Age patterns of severe paediatric malaria and their relationship to Plasmodium falciparum transmission intensity. Malar J 8: 4.

64. Snow RW, Marsh K (2002) The consequences of reducing transmission of *Plasmodium falciparum* in Africa. Adv Parasitol 52: 235-264.

65. Smith TA, Leuenberger R, Lengeler C (2001) Child mortality and malaria transmission intensity in Africa. Trends Parasitol 17: 145-149.

66. Smith T, Killeen G, Lengeler C, Tanner M (2004) Relationships between the outcome of *Plasmodium falciparum* infection and the intensity of transmission in Africa. Am J Trop Med Hyg 71: 80-86.

67. Ross A, Smith T (2006) The effect of malaria transmission intensity on neonatal mortality in endemic areas. Am J Trop Med Hyg 75: 74-81.

68. Greenwood B, Alonso P, ter Kuile FO, Hill J, Steketee RW (2007) Malaria in pregnancy: priorities for research. Lancet Infect Dis 7: 169-174.

69. Haghdoost AA, Alexander N, Smith T (2007) Maternal malaria during pregnancy and infant mortality rate: critical literature review and a new analytical approach. J Vector Borne Dis 44: 98-104.

70. Tagbor H, Bruce J, Browne E, Greenwood B, Chandramohan D (2008) Malaria in pregnancy in an area of stable and intense transmission: is it asymptomatic? Trop Med Int Health 13: 1016-1021.

71. Clerk CA, Bruce J, Greenwood B, Chandramohan D (2009) The epidemiology of malaria among pregnant women attending antenatal clinics in an area with intense and highly seasonal malaria transmission in northern Ghana. Trop Med Int Health 14: 688-695.

72. Ghani AC, Sutherland CJ, Riley EM, Drakeley CJ, Griffin JT, et al. (2009) Loss of population levels of immunity to malaria as a result of exposure-reducing interventions: consequences for interpretation of disease trends. PLoS ONE 4: e4383.

73. Gatton ML, Cheng Q (2004) Modeling the development of acquired clinical immunity to Plasmodium falciparum malaria. Infect Immun 72: 6538-6545.

74. Martens WJ (1995) Climate change and malaria: exploring the risks. Med War 11: 202-213.

75. Martens WJ, Niessen LW, Rotmans J, Jetten TH, McMichael AJ (1995) Potential impact of global climate change on malaria risk. Environ Health Perspect 103: 458-464.

76. Guerra CA, Gikandi PW, Tatem AJ, Noor AM, Smith DL, et al. (2008) The limits and intensity of *Plasmodium falciparum* transmission: implications for malaria control and elimination worldwide. PLoS Med 5: e38.

77. Guerra CA, Howes RE, Patil AP, Gething PW, Van Boeckel TP, et al. (2010) The international limits and population at risk of Plasmodium vivax transmission in 2009. PLoS Negl Trop Dis 4: e774.

78. Bayoh MN, Lindsay SW (2003) Effect of temperature on the development of the aquatic stages of Anopheles gambiae sensu stricto (Diptera: Culicidae). Bull Entomol Res 93: 375-381.

79. Bayoh MN, Lindsay SW (2004) Temperature-related duration of aquatic stages of the Afrotropical malaria vector mosquito Anopheles gambiae in the laboratory. Med Vet Entomol 18: 174-179.

80. Hay SI, Snow RW, Rogers DJ (1998) Predicting malaria seasons in Kenya using multitemporal meteorological satellite sensor data. Trans R Soc Trop Med Hyg 92: 12-20.

81. Kilian AH, Langi P, Talisuna A, Kabagambe G (1999) Rainfall pattern, El Nino and malaria in Uganda. Trans R Soc Trop Med Hyg 93: 22-23.

82. Teklehaimanot HD, Lipsitch M, Teklehaimanot A, Schwartz J (2004) Weather-based prediction of Plasmodium falciparum malaria in epidemic-prone regions of Ethiopia I. Patterns of lagged weather effects reflect biological mechanisms. Malar J 3: 41.

83. Gething PW, Smith DL, Patil AP, Tatem AJ, Snow RW, et al. (2010) Climate change and the global malaria recession. Nature 465: 342-345.

84. Carter R (2002) Spatial simulation of malaria transmission and its control by malaria transmission blocking vaccination. Int J Parasitol 32: 1617-1624.

85. Smith DL, Dushoff J, McKenzie FE (2004) The risk of a mosquito-borne infection in a heterogeneous environment. PLoS Biol 2: e368.

86. Cosner C, Beier JC, Cantrell RS, Impoinvil D, Kapitanski L, et al. (2009) The effects of human movement on the persistence of vector-borne diseases. J Theor Biol 258: 550-560.

87. Smith DL, McKenzie FE (2004) Statics and dynamics of malaria infection in Anopheles mosquitoes. Malar J 3: e13.

88. Killeen GF, Smith TA, Ferguson HM, Mshinda H, Abdulla S, et al. (2007) Preventing childhood malaria in Africa by protecting adults from mosquitoes with insecticide-treated nets. PLoS Med 4: e229.

89. Killeen GF, Smith TA (2007) Exploring the contributions of bed nets, cattle, insecticides and excitorepellency to malaria control: a deterministic model of mosquito host-seeking behaviour and mortality. Trans R Soc Trop Med Hyg 101: 867-880.

90. Le Menach A, Takala S, McKenzie FE, Perisse A, Harris A, et al. (2007) An elaborated feeding cycle model for reductions in vectorial capacity of night-biting mosquitoes by insecticide-treated nets. Malar J 6: 10.

91. Okell LC, Drakeley CJ, Bousema T, Whitty CJ, Ghani AC (2008) Modelling the impact of artemisinin combination therapy and long-acting treatments on malaria transmission intensity. PLoS Med 5: e226; discussion e226.

92. Lawpoolsri S, Klein EY, Singhasivanon P, Yimsamran S, Thanyavanich N, et al. (2009) Optimally timing primaquine treatment to reduce Plasmodium falciparum transmission in low endemicity Thai-Myanmar border populations. Malar J 8: 159.
