# Supplementary material for: A Research Agenda for Malaria Eradication: Modeling
Source: PLoS Med. 2011 Jan 25;8(1):e1000403. doi: 10.1371/journal.pmed.1000403 (PMC3026697; doi:10.1371/journal.pmed.1000403)
Supplement: Text S2 — Harmonization of databases; annex of existing databases. (0.27 MB DOC) [file pmed.1000403.s002.doc]

**Annex 2. Harmonization of databases – annex of existing databases**

Table 1. Examples of databases that might be included in the assemblage.

|  | **Type of data** | **Example outcomes** | **Ownership** | **Access** | **Outstanding issues** |
| --- | --- | --- | --- | --- | --- |
| Malaria therapy [1,2] | Daily sequences of parasite densities and of infectiousness to mosquitoes in therapeutic malaria cases | Within-host dynamics | In the public domain. | Yes | Different versions of the data exist because the records contain inconsistencies |
|  |  | Duration of infectiousness |  |  |  |
| Garki project (Nigeria) [3] | Individual parasitological, entomological and intervention data from field trials of IRS (indoor residual spraying) and mass treatment | EIR vs. PR by age | WHO | Yes | Can be downloaded from |
|  |  | Parasite densities by age and EIR |  |  | <http://www.swisstph.ch/research/public-health-and-epidemiology/biostatistics/downloads.html> |
| Dielmo and Ndiop longitudinal studies (Senegal) [4,5,6,7,8] | Individual parasitological, entomological and clinical data | EIR vs PR by age | Institut de Recherche pour le Développement France | No |  |
|  |  | Parasite densities by age and EIR |  |  |  |
|  |  | Clinical incidence by age and EIR |  |  |  |
| Wosera malaria projects (PNG) [9,10,11,12] | Individual parasitological, entomological and clinical data from observational studies and field trials | PR, clinical incidence, intervention coverage, etc. by age and EIR | PNG Institute of Medical Research | Not yet | Complex funding; contains individual records, raising issues of confidentiality |
| Kilombero Malaria Project (Tanzania)  [13,14,15] | Individual parasitological, entomological and clinical data from observational studies and field trials | Prevalence, clinical incidence, intervention coverage, etc. by age and EIR for a defined area. | Swiss Tropical and Public Health Institute and Ifakara Health Institute | Not yet | Complex funding; contains individual records, raising issues of confidentiality |
| MTIMBA (Malaria Transmission Intensity and Mortality Burden Across Africa) [demographic surveillance system (DSS) sites] (unpublished) | Geolocated entomological and demographic (births, deaths) data from DSSs | Mortality by age and EIR for specific sites | DSS sites | No | Can be linked to demographic, economic, morbidity, environmental data, etc. |
|  |  |  |  |  | Ownership and access issues for the overall DSS databases are complex, and currently an active area of discussion. |
| CHERG (Child Health Epidemiology Reference Group) malaria morbidity dataset [16] |  | Morbidity by age and EIR | CHERG group | No |  |
| Navrongo genotyping studies (Saturn)  [17,18] | Repeated cross-sectional surveys and msp-2 genotyping | Multiplicity of infection by age and EIR | Navrongo Health Research Centre/Swiss TPH | Not yet |  |
|  |  | Transition rates for individual infections |  |  |  |
| MAP (Malaria Atlas Project) [19,20,21,22,23] | Global assemblages of prevalence and vector species data | Georeferenced PR |  | Yes | http://www.map.ox.ac.uk/ |
|  |  | Geo-referenced vector species |  |  |  |
| MARA (Mapping Malaria Risk in Africa) [24,25] | Africa-wide assemblage of prevalence data; various malaria maps produced from climatic suitability models and/or analysis of prevalence data | Geo-referenced parasite prevalence |  | Yes | http://www.mara-database.org |
| Demographic and Health Surveys (DHS )  (contain intervention data) | Representative household surveys, including malaria intervention data | Geo-referenced intervention and contextual determinants | Macro International; open access | Yes |  |
| Malaria indicator surveys (MISs) | Representative national-level surveys, including malarialogical indicators in children | Geo-referenced parasite prevalence and intervention coverage | MACEPA (Malaria Control and Evaluation Partnership in Africa), National Malaria Control Programs (NMCPs), Macro International |  |  |
| Malaria maps from national malaria control programmes  (NMCPs) | Heterogeneous data depending on the country | Clinical incidence by geographical area | NMCPs | Yes |  |
| WWARN (WorldWide Antimalarial Resistance Network; drug-resistance monitoring) [26,27] |  | Geo-referenced drug resistance rates, including molecular markers | WWARN network | Yes | Recently initiated project |
| WHO malaria database [28] | National-level incidence data and contextual information | API, annual parasite incidence and contextual information by country | WHO | Yes | Detailed information is very incomplete; standard information about each country can be extracted from the World Malaria Report [16] |
| Clinical databases | Assemblages of API/clinical incidence disaggregated by time period and location | Clinical incidence and incidence of severe disease by age | ? | Not yet | Not clear that anyone is compiling these; a clear gap in the available databases |

**REFERENCES:**

1. Collins WE, Jeffery GM (1999) A retrospective examination of the patterns of recrudescence in patients infected with Plasmodium falciparum. Am J Trop Med Hyg 61: 44-48.

2. Collins WE, Jeffery GM (2003) A retrospective examination of mosquito infection on humans infected with Plasmodium falciparum. Am J Trop Med Hyg 68: 366-371.

3. Molineaux L, Gramiccia G (1980) The Garki Project. Geneva: World Health Organization.

4. Rogier C, Trape JF (1995) [Study of premunition development in holo- and meso-endemic malaria areas in Dielmo and Ndiop (Senegal): preliminary results, 1990-1994[]. Med Trop (Mars) 55: 71-76.

5. Fontenille D, Lochouarn L, Diatta M, Sokhna C, Dia I, et al. (1997) Four years' entomological study of the transmission of seasonal malaria in Senegal and the bionomics of Anopheles gambiae and A. arabiensis. Trans R Soc Trop Med Hyg 91: 647-652.

6. Fontenille D, Lochouarn L, Diagne N, Sokhna C, Lemasson JJ, et al. (1997) High annual and seasonal variations in malaria transmission by anophelines and vector species composition in Dielmo, a holoendemic area in Senegal. Am J Trop Med Hyg 56: 247-253.

7. Zwetyenga J, Rogier C, Spiegel A, Fontenille D, Trape JF, et al. (1999) A cohort study of Plasmodium falciparum diversity during the dry season in Ndiop, a Senegalese village with seasonal, mesoendemic malaria. Trans R Soc Trop Med Hyg 93: 375-380.

8. Cancre N, Tall A, Rogier C, Faye J, Sarr O, et al. (2000) Bayesian analysis of an epidemiologic model of Plasmodium falciparum malaria infection in Ndiop, Senegal. Am J Epidemiol 152: 760-770.

9. Genton B, Hii J, al-Yaman F, Paru R, Beck HP, et al. (1994) The use of untreated bednets and malaria infection, morbidity and immunity. Ann Trop Med Parasitol 88: 263-270.

10. Genton B, al-Yaman F, Beck HP, Hii J, Mellor S, et al. (1995) The epidemiology of malaria in the Wosera area, East Sepik Province, Papua New Guinea, in preparation for vaccine trials. I. Malariometric indices and immunity. Ann Trop Med Parasitol 89: 359-376.

11. Smith T, Genton B, Baea K, Gibson N, Narara A, et al. (2001) Prospective risk of morbidity in relation to malaria infection in an area of high endemicity of multiple species of Plasmodium. Am J Trop Med Hyg 64: 262-267.

12. Smith T, Genton B, Betuela I, Rare L, Alpers MP (2002) Mosquito nets for the elderly? Trans R Soc Trop Med Hyg 96: 37-38.

13. Smith T, Charlwood JD, Kihonda J, Mwankusye S, Billingsley P, et al. (1993) Absence of seasonal variation in malaria parasitaemia in an area of intense seasonal transmission. Acta Trop 54: 55-72.

14. Charlwood JD, Kihonda J, Sama S, Billingsley PF, Hadji H, et al. (1995) The rise and fall of Anopheles arabiensis (Diptera: Culicidae) in a Tanzanian village. Bulletin of Entomological Research 85: 37-44.

15. Smith T, Hurt N, Teuscher T, Tanner M (1995) Is fever a good sign for clinical malaria in surveys of endemic communities? Am J Trop Med Hyg 52: 306-310.

16. Bryce J, Boschi-Pinto C, Shibuya K, Black RE (2005) WHO estimates of the causes of death in children. Lancet 365: 1147-1152.

17. Owusu-Agyei S, Smith T, Beck HP, Amenga-Etego L, Felger I (2002) Molecular epidemiology of Plasmodium falciparum infections among asymptomatic inhabitants of a holoendemic malarious area in northern Ghana. Trop Med Int Health 7: 421-428.

18. Sama W, Owusu-Agyei S, Felger I, Dietz K, Smith T (2006) Age and seasonal variation in the transition rates and detectability of Plasmodium falciparum malaria. Parasitology 132: 13-21.

19. Guerra CA, Hay SI, Lucioparedes LS, Gikandi PW, Tatem AJ, et al. (2007) Assembling a global database of malaria parasite prevalence for the Malaria Atlas Project. Malar J 6: 17.

20. Guerra CA, Gikandi PW, Tatem AJ, Noor AM, Smith DL, et al. (2008) The limits and intensity of *Plasmodium falciparum* transmission: implications for malaria control and elimination worldwide. PLoS Med 5: e38.

21. Hay SI, Guerra CA, Gething PW, Patil AP, Tatem AJ, et al. (2009) A world malaria map: *Plasmodium falciparum* endemicity in 2007. PLoS Med 6: e1000048.

22. Guerra CA, Howes RE, Patil AP, Gething PW, Van Boeckel TP, et al. (2010) The international limits and population at risk of Plasmodium vivax transmission in 2009. PLoS Negl Trop Dis 4: e774.

23. Hay SI, Sinka ME, Okara RM, Kabaria CW, Mbithi PM, et al. (2010) Developing global maps of the dominant anopheles vectors of human malaria. PLoS Med 7: e1000209.

24. Le Sueur D, Binka F, Lengeler C, De Savigny D, Snow B, et al. (1997) An atlas of malaria in Africa. Afr Health 19: 23-24.

25. Craig MH, Snow RW, le Sueur D (1999) A climate-based distribution model of malaria transmission in sub-Saharan Africa. Parasitol Today 15: 105-111.

26. Price RN, Dorsey G, Ashley EA, Barnes KI, Baird JK, et al. (2007) World Antimalarial Resistance Network I: clinical efficacy of antimalarial drugs. Malar J 6: 119.

27. Bacon DJ, Jambou R, Fandeur T, Le Bras J, Wongsrichanalai C, et al. (2007) World Antimalarial Resistance Network (WARN) II: in vitro antimalarial drug susceptibility. Malar J 6: 120.

28. WHO (2009) World Malaria Report 2009. Geneva, Switzerland: World Health Organization.
