# Supplementary material for: A Research Agenda for Malaria Eradication: Modeling
Source: PLoS Med. 2011 Jan 25;8(1):e1000403. doi: 10.1371/journal.pmed.1000403 (PMC3026697; doi:10.1371/journal.pmed.1000403)
Supplement: Text S3 — Interface for users and cyberinfrastructure. (0.18 MB DOC) [file pmed.1000403.s003.doc]

**S 3. Interface for users and cyberinfrastructure**


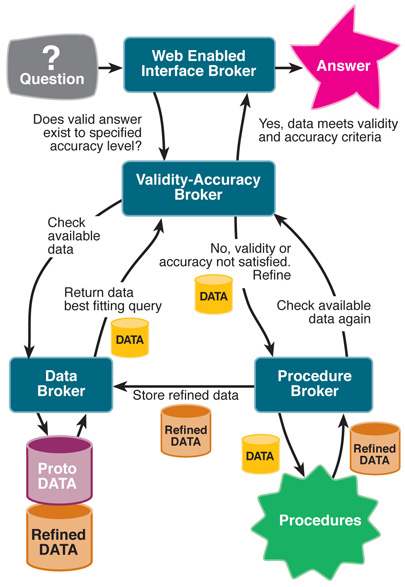


**Figure A1. User interface and cyberinfrastructure.**

As shown in Figure A1 (above), the system is invoked in response to a user’s question, which is accompanied by implicit or explicit minimum standards the answer must meet, such as timeliness, accuracy and geographic specificity. The system analyzes the question to determine which combination of models acting on what data is required to answer it. This analysis is made possible by an ontology that describes the relationships of the models, the data and common questions. A data broker determines whether the requisite data are already available. Data will be deemed available if they reside in an accessible database already or if they can be synthesized by a (predictive) model from available data or if they can be interpolated with acceptable accuracy from available data. If a critical link in the process is unavailable, the user will be informed and possible surrogates will be suggested. For example, if the question is to compare two possible intervention strategies in a particular region, but no prevalence data specific to that region are available, the system would inform the user and suggest using prevalence data from the nearest region where they are available. If the question requires a model or series of models to be run, the system will determine available computational resources, stage required data, invoke the models and collect the outputs. At this point, the system may decide to store the output if it would be expensive to recompute. The system may call on associated tools to summarize the collected output into a form most useful for answering the question.

Output of analyses performed by the structure will include a comprehensive list of citations of the source materials. A list of caveats (provided by the stakeholders) on the scope of appropriate use will also be included. This provides the stakeholders with an incentive to include their information in the system with the assurance that results will not be misinterpreted.

The proposed system is conceptually divided into three layers:

1. An interface layer contains the user interfaces to the system. Components such as GUIs (graphical user interfaces), scripting tools and visualization tools belong to this layer. These tools may be generic, such as data-exploration tools, or domain-specific, such as a tool for calibrating the parameters of transmission models. These tools may run on a variety of computing hardware, ranging from cell phones and other handheld devices, through laptop and desktop computers, to high-end visualization theaters with 3D immersive environments.
2. A query layer provides an abstraction between the interface layer and the physical layer. Its main job is to match user requirements to system capabilities in a way that isolates the user from the details of the physical layer.
3. The physical layer consists of a hardware (sensor) grid, a data grid, a computational grid and a model grid, explained below. These are the components of the system that do the actual work. Attention is given to maintaining information security, personal privacy and anonymity.

The basic architecture consists of the following components:

- 1. sensor and data fusion grid: a data-injection system to incorporate online (e.g. sensors) and offline (e.g. historical databases) data;
  2. data-management grid: scalable data management and movement architecture, and a system to support model execution and analytics;
  3. coordinating architecture: a distributed coordinating architecture for information fusion, model execution and data processing;
  4. modeling grid: scalable models of physical, biological and social processes;
  5. decision-informatics and analytics grid: scalable methods for visual and data analytics to support analysts;
  6. compute grid: physical resources on which computations take place.

Several research issues will need to be addressed in building the cyberinfrastructure for malERA:

- Interoperability of different computational resources such as clouds and grids must be understood.
- Information services for managing the sensor collections must be defined.
- Reliability and virtualization of the components’ interconnections must be addressed.
- Metadata and provenance of transformed data must be defined and managed.
- Complicated failure detection and recovery strategies must be addressed.
- End-to-end security of the messages and their recipients at many levels (authentication, authorization, privacy, etc.) across a heterogeneous system must be researched.

Here we briefly discuss the coordinating architecture. It serves the role of coordinating the diverse simulations, data management tools, visual and data analytic tools, and end users. It is organized around the software as a service (SaaS) paradigm. An example of such a cyberinfrastructure, Simfrastructure [[[1]](#footnote-2)], uses Javaspaces as the implementing construct, but the basic concepts are generic and readily implemented using other similar technologies. The asynchronous ensembles in its architecture consist of simulation models, databases, GUIs and analytical tools. The basic concept is that of brokers – coordinating processes responsible for achieving a desired workflow by asynchronously invoking appropriate ensembles – as sketched in A1. They are lightweight processes that are assigned the task of requesting information from various ensembles and communicating between ensembles. Brokers use associative memory, or blackboards, for communicating software objects between them. For computational efficiency and security these blackboards are in general distributed and organized hierarchically.

Brokers are also organized hierarchically. This hierarchy captures calling rules. Brokers post appropriate objects on the blackboard. These software objects can represent queries (i.e. requests for services), offers of service and the results of queries. This architecture uses the generative communication paradigm; brokers act as coordinators for these purposes. Brokers are responsible for understanding what information needs to be communicated between various asynchronous ensembles. In the architecture envisioned here, achieving a given functionality must take into account computational efficiency, memory requirements and accuracy: brokers call appropriate computation and evaluation processes to conclude whether the object returned conforms to the required specification. Simfrastructure uses three classes of brokers:

1. Edge brokers mediate access to particular sensors (models, data, services, etc.), removing the need for the resource to communicate directly with any other resource.
2. Service brokers implement a workflow that coordinates the edge brokers in response to satisfy a given query.
3. Coordination brokers coordinate the overall workflow, partition the solution space and serve as the service discovery mechanism.

Brokers will in general not know how a specific request can be satisfied, and therefore use a blackboard as a way to broadcast their requests. Brokers that can invoke appropriate processes fulfill these requests by first marking the request as being processed, and then placing the result (or, in the case of a large result, the location at which the result can be retrieved) onto the blackboard. The broker requests are highly asynchronous; in general, requests are generated on demand when a specific analysis needs to be done by an analyst. At that time, the cyberinfrastructure has very little control over the specific computing and data resources at its disposal. Broker-based architecture allows us to develop solutions that protect participating institutions’ intellectual property.

The flexibility of a collaborative environment will enable deployment of uniform technology from tactical to strategic levels. Furthermore, it will combine in a unified scalable interface (i) the advanced analytics capabilities needed by expert analysts accessing high-performance resources; (ii) simplified task-specific views for field operatives using low-end resources such as handheld devices; and (iii) high-quality, focused summaries as actionable intelligence for decision makers. At the interface level, we will envision information-visualization techniques for large-scale dynamic geospatial information that current technologies, such as Environmental Systems Research Institute and Google Earth, do not adequately provide and are not expected to develop at the necessary scale. For example, we anticipate interactively visualizing and updating uncertainty metrics while a query is being resolved, enabling quick, informed decisions by the user. Furthermore, cyberinfrastructure research should include studying a range of quality-for-speed tradeoffs in the data presentation to accommodate various activities (e.g. monitoring, data exploration, presentation and communication in collaborative sessions). The visualization should be tightly coupled with development of the spatiotemporal indexing and query capabilities to maximize the ability to compute and present the results in a progressive hypothesis-driven environment.

1. ?. Atkins K, Barrett C, Beckman R, Bisset K, Chen J, et al. (2008) An interaction based composable architecture for building scalable models of large social, biological, information and technical systems. CTWatch Quart 4: 46-53. [↑](#footnote-ref-2)
